# Erratum

**DOI:** 10.5935/abc.20180087

**Published:** 2018-05

Edition of December 2017, vol. 109 (6), Supl. 2, p. 1-34

In the “Atualização das Diretrizes Brasileiras de Valvopatias: Abordagem das Lesões Anatomicamente Importantes”, published as a supplement to the Arquivos Brasileiros de Cardiologia [Arq Bras Cardiol 2017; 109(6Supl.2):1-34], the following corrections should be considered:

- In table 18, column 5, line 6: replacement of “IIb” with “IIb C”.
- In Table 24, line 4: the items “IB, IB, IC” should be aligned with the phrase” Fração de ejeção < 50%”. In line 5, items “IIa, IIa B, I C” should be aligned with the phrase “ Ausência de reserva inotrópica no teste ergométrico e/ou baixa capacidade funcional”. On line 6, remove item “B”.
- In table 24, section “Tratamento cirúrgico convencional”, change from “Sem reserva contrátil” to “Sem reserva contrátil + escore de cálcio valvar elevado”.
- In table 43, section “ TAVI ritmo sinusal “, change of recommendations as follows:
  Varfarina – Correct: ESC - IIb B*.
  AAS + clopidogrel (6 meses, seguido por AAS por período indefinido) – Correct: SBC - IIa C; AHA - IIb C; ESC - IIa C. NOACs – Correct: SBC – III.
- Inclusion of the name of Dr. Samira Kaissar Nasr Ghorayeb in the document.

Edition of April 2016, vol. 106 (4), Supl. 1, p. 1-23

In the “III Diretrizes da Sociedade Brasileira de Cardiologia sobre Análise e Emissão de Laudos Eletrocardiográficos”, published as a supplement to the Arquivos Brasileiros de Cardiologia [Arq Bras Cardiol 2016; 106(4Supl.1):1-23], the following corrections should be considered:

On page 1, section 2.1., text correction “O eixo de P pode variar entre -30º e +90º” to “O eixo de P pode variar entre 0º e +90º”.

